# Systematic review and metanalysis in urology: how to interpret the forest plot

**DOI:** 10.1590/S1677-5538.IBJU.2023.9911

**Published:** 2024-02-07

**Affiliations:** 1 Universidade do Estado do Rio de Janeiro Unidade de Pesquisa Urogenital Rio de Janeiro RJ Brasil Unidade de Pesquisa Urogenital - Universidade do Estado do Rio de Janeiro - Uerj, Rio de Janeiro, RJ, Brasil;; 2 Hospital Federal da Lagoa Rio de Janeiro RJ Brasil Serviço de Urologia, Hospital Federal da Lagoa, Rio de Janeiro, RJ, Brasil

## COMMENT

The systematic review follows rigid rules to find the best scientific evidence. This kind of publication aims to bring evidence together to answer a pre-defined research question. The research needs to make a search in all available databases. Systematic reviews should include a synthesis of the data that have been found. Data synthesis can involve summarizing quantitative and/or qualitative findings (1). Systematic reviews of quantitative data may include meta-analysis. The analysis of quantitative data in meta-analysis is the key to interpret the results of the study (1). In this comment we show the most important topics in the Forest plot interpretation.

**Figure 1:**
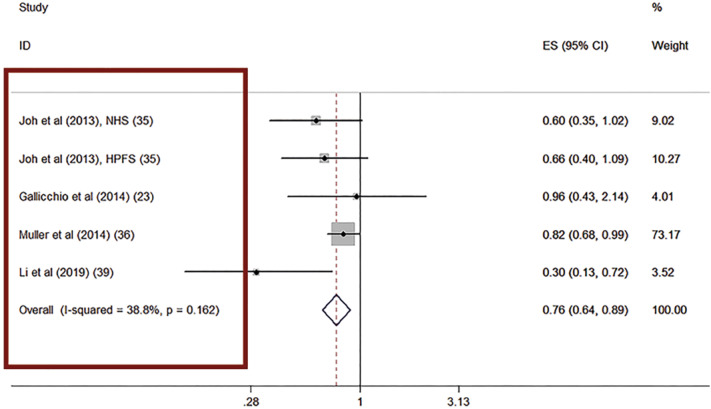
The figure shows the quantitative data in a meta-analysis. In the left column (red square) we can observe the author's name of the studies included in meta-analysis.

When studying we must be familiar with the various types of graphs used in a meta-analysis. In the left column of a quantitative data the author's name of the studies included in meta-analysis are registered (Figure-1) (2).

**Figure 2:**
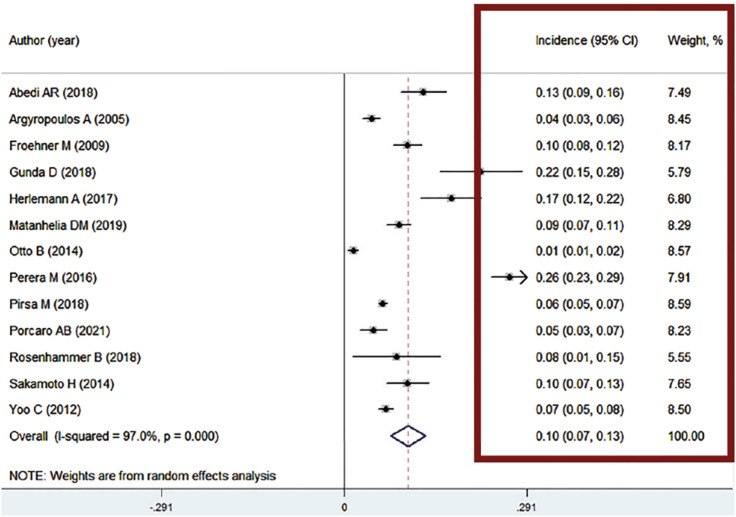
The figure shows the quantitative data in a meta-analysis. In the right column (red square) we can observe the confidence intervals (CI) and the weights assigned to each study.

**Figure 3:**
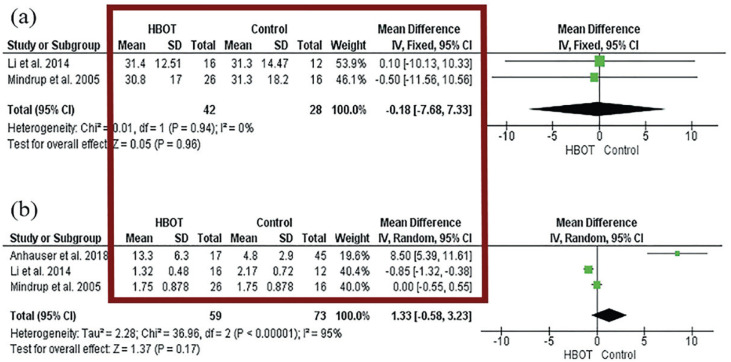
The figure shows the quantitative data in a meta-analysis. In column highlighted by the red square we can observe the mean, standard deviation (SD) and the mean difference assigned to each group studied in meta-analysis (2).

In the right column of the figure 2 we can observe the confidence intervals (CI) registered in the quantitative data of a meta-analysis. Some forest plots may also provide information about the weights assigned to each study or independent variable in the analysis (Figure-2). These weights can be represented by the size of the "boxes" on the plot or by a number associated with each study. In some situations, the authors can include the values of the mean, standard deviation (SD) and the mean difference assigned to each group studied in meta-analysis in the center of the graphic (Figure-3) (3).

**Figure 4:**
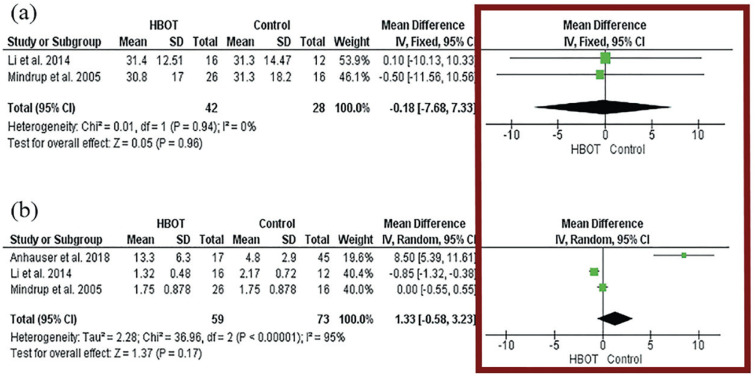
The figure shows the quantitative data in a meta-analysis – the Forest plot. In the column highlighted by the red square we can observe the vertical axis of the forest plot (independent variables or individual studies included in the analysis) and the horizontal axis (represents the outcome measure or estimated effect). The vertical lines represent the confidence intervals for each study or independent variable. The central line in each "box" represents the point estimate for each study or independent variable. If the confidence intervals of different lines do not overlap, it suggests that there is a statistically significant difference between the represented studies or independent variables. If the lines cross the null vertical line (typically a vertical line at a value of 1 or 0), it indicates that there is no statistically significant difference between the compared interventions or independent variables (2).

The vertical axis of the forest plot typically represents the independent variables or individual studies included in the analysis. Each study or independent variable is represented by a line on the plot (Figure-4). The horizontal axis usually represents the outcome measure or estimated effect. It can be a measure of risk, such as odds ratio or hazard ratio, or a measure of difference, such as mean difference or proportion difference (Figure-4). The vertical lines represent the confidence intervals for each study or independent variable. The length of the line indicates the precision of the estimate. The longer the line, the less precise the estimate. The central line in each "box" represents the point estimate for each study or independent variable. It can be the point estimate or the weighted average of the studies (Figure 4). If the confidence intervals of different lines do not overlap, it suggests that there is a statistically significant difference between the represented studies or independent variables. If the lines cross the null vertical line (typically a vertical line at a value of 1 or 0), it indicates that there is no statistically significant difference between the compared interventions or independent variables (Figure-4).

**Figure 5:**
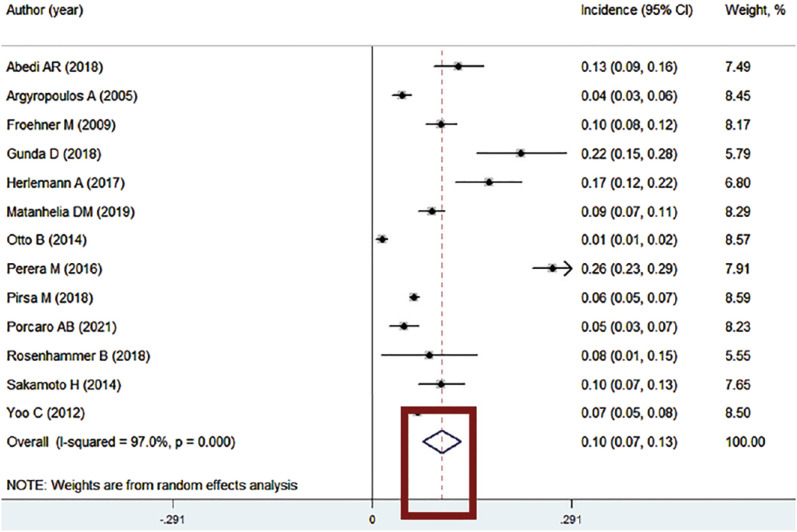
The figure shows the Diamond in a Forest plot. The diamond represents the value of each study (size determined by 95% CI). If the diamond is not touching the vertical line represents that the final result is statistically significant. If the diamond touches the line represents the studies do not have statistical significance.

The value of each study depends on the sample size and precision, which will receive weight values. These weight values will be evaluated to compound the diamond represent the final result. Diamond size determined by 95% CI if not touching the vertical line represents that the final result is statistically significant (left – better intervention/ Right – better control group) If the diamond touches the line represents the studies do not have statistical significance (Figure-5).

The legends and annotations in the plot may provide additional information about the studies or independent variables included in the analysis, the outcome measures used, and other relevant statistics. Remember that the precise interpretation of the forest plot may depend on the specific context of the study or analysis. If you have any doubts about a specific forest plot, it's always advisable to consult the cited references or seek the assistance of an expert in the field.

## References

[1] Gough D, Oliver S, Thomas J (2012). An introduction to systematic reviews.

[2] Wu J, Yang N, Yuan M (2021). Dietary and circulating vitamin D and risk of renal cell carcinoma: a meta-analysis of observational studies. Int Braz J Urol.

[3] Guo Z, He J, Huang L, Wang Z, Hu P, Wang S (2022). Prevalence and risk factors of incidental prostate cancer in certain surgeries for benign prostatic hyperplasia: A systematic review and meta-analysis. Int Braz J Urol.

